# Carbon Quantum Dots: Synthesis, Characteristics, and Quenching as Biocompatible Fluorescent Probes

**DOI:** 10.3390/bios15020099

**Published:** 2025-02-10

**Authors:** Arif Kamal, Seongin Hong, Heongkyu Ju

**Affiliations:** 1 Department of Physics, Gachon University, Seongnam-si 13120, Republic of Korea; arifkamal37@gachon.ac.kr; 2 Gachon Bionano Research Institute, Gachon University, Seongnam-si 13120, Republic of Korea; 3 Department of Semiconductor Engineering, Gachon University, Seongnam-si 13120, Republic of Korea

**Keywords:** carbon dots, quantum dots, synthesis, biocompatibility, fluorescence quenching, photoluminescence

## Abstract

Carbon quantum dots (CQDs), a new class of carbon-based nanomaterials, have emerged as nano-scaled probes with photoluminescence that have an eco-friendly and bio-compatible nature. Their cost-efficient synthesis and high photoluminescence quantum yields make them indispensable due to their application in opto-electronic devices, including biosensors, bioimaging, environmental monitoring, and light sources. This review provides intrinsic properties of CQDs such as their excitation-dependent emission, biocompatibility, and quenching properties. Diverse strategies for their easy synthesis are divided into bottom-up and top-down approaches and detailed herein. In particular, we highlight their luminescence properties, including quenching mechanisms that could even be utilized for the precise and rapid detection of biomolecules. We also discuss methodologies for the mitigation of fluorescence quenching, which is pivotal for the application of CQDs in biosensors and bioimaging.

## 1. Introduction

**Figure 1:**
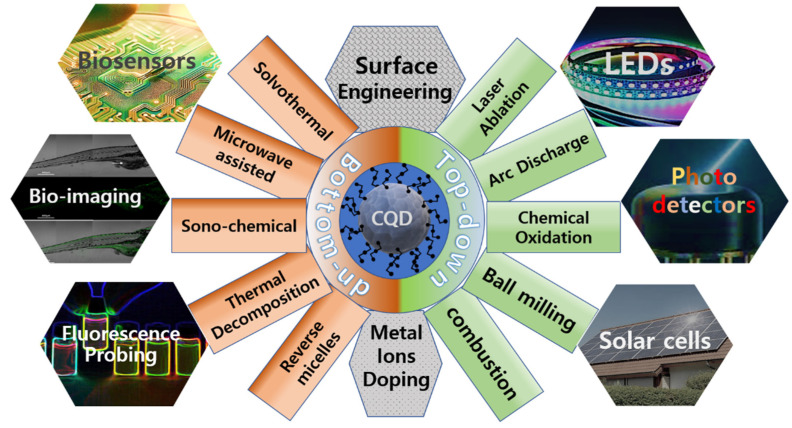
Synthesis and potential applications of carbon quantum dots (CQDs).

**Figure 2:**
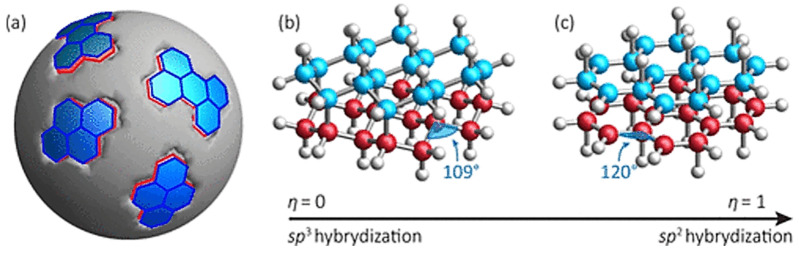
(**a**) Schematic representation of a CQD core (grey) and its small domains made of fully (**b**) sp3- and (**c**) sp2-hybridized carbon atoms. Blue and red spheres are C atoms, and white spheres are H atoms. The angle between the C–C bonds is 109.5° in (**b**) and 120° in (**c**). Reprinted with permission from reference [2]. Copyright 2019 American Chemical Society.

Carbon-based quantum dots (CQDs), often referred to as carbon nano-dots [1], are a new class of vividly fluorescent nano-particles (sizes of less than 10 nm). They can be synthesized by numerous approaches for applications such as biosensors/bioimaging, photodetectors and solar cells, as summarized in Figure 1. CQDs are known to have an amorphous core, as well as inner domains of partially sp^2^- and sp^3^- hybridized carbon atoms (Figure 2) [2]. A variety of oxygen-rich functional groups can also be chemically immobilized on their surface, permitting surface engineering for various binding and conjugation applications [3,4]. The nano-scale particle size of the CQDs basically allows for harnessing a quantum confinement effect of excited carriers [5], while both the presence of hybridized domains and the availability of various surface functional groups can manipulate surface energy traps. This enables CQDs to absorb electromagnetic radiation, with their spectral range extending into ultraviolet and visible regions. A radiative decay [6] of excitons generated by absorptions produces an emission of photons at wavelengths longer than that of the absorption. For CQDs, high photoluminescence quantum yields (PLQYs) have been reported to be up to 99% [7].

CQDs were first discovered by Xu et al. in 2004, during the purification of single walled carbon nanotubes (SWCNTs) [7]. They were reported to have ellipsoidal carbon cores (53.93%) with carboxyl functional groups on their surface (40% O, 2.56% H, and 1.20% N by EDAX analysis). The estimated average size of CQDs was less than 18 nm, while CQDs emitted multi-color luminescence with a small PLQY, e.g., 1.6% at a photoexcitation wavelength of 366 nm. Later on, Ya-Ping et al., in 2006, reported the first synthesis of CQDs through the laser ablation of carbon and their surface passivation with organic molecules like PEG1500N. They fluoresced with blue, green, and red emissions at a 400 nm excitation wavelength. Their non-blinking bright emission and stable PLQYs of over 10% were attributed to their surface functionalities and a quantum confinement effect of electrons and holes in CQDs [8]. To date, significant efforts have been put into the synthesis of CQDs of variable size, the spectral tunability of their fluorescence, and enhancing PLQYs [9].

Besides the natural abundance of their raw precursors and their cost-bearable synthesis, CQDs are eco-friendly and bio-compatible nano-particles [10,11] as compared to their analogous materials, toxic metal-based quantum dots (QDs) and organic dyes. In addition to these unique features, their high color purity, tunable fluorescence, and high quantum yields permit CQDs to hold significant potential for use in a wide variety of devices such as biosensors [12], light-emitting diodes [13,14,15], photodetectors [16], solar cells [17], anti-counterfeiting ink with encrypted information [18], electro-luminescent light-emitting diodes (ELEDs) [19], CO_2_ reduction [20], and antibacterial applications [21]. CQDs, however, face severe limitations such as fluorescence quenching that can occur quite often when they are subject to ambient stimuli. For example, when CQDs are deposited on metallic films, the radiative decay of their excitons is deactivated, reducing their fluorescence. Some CQDs are fluorescently quenched when they are dried out from their solvent form into powder. While these properties could hinder CQDs from being used in optical devices, their quenching features could, on the other hand, be utilized for detecting biomolecules that are brought into their proximity [22,23,24,25,26].

In this review article, we provide insight into the optical properties of CQDs and a variety of methods for their synthesis. Particularly, we discuss the fluorescence quenching mechanisms of CQDs, together with conceivable approaches to utilize fluorescence quenching for various types of molecular detection in biosensors and the strategies to mitigate their fluorescence quenching for use in opto-electronic devices.

## 2. Synthesis of Carbon Quantum Dots

**Figure 3:**
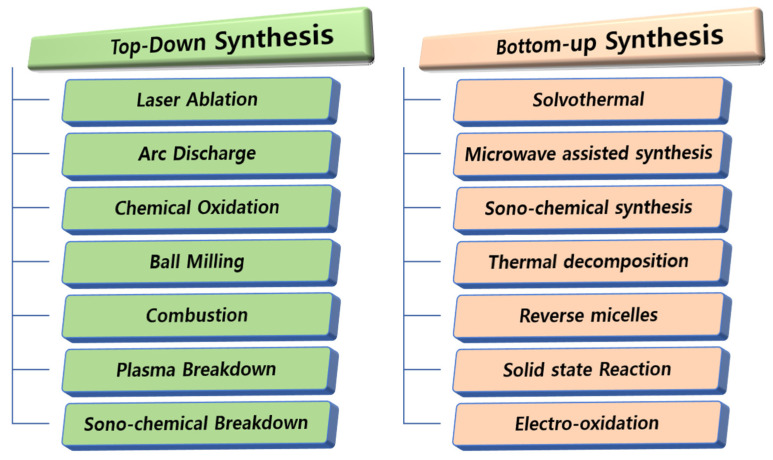
CQD synthesis methods: top-down (**left**) and bottom-up (**right**) approaches.

Numerous techniques have been reported for the synthesis of CQDs that allow size controllability and use various surface chemistries to tailor their physicochemical properties. CQDs can be directly synthesized by breaking down the raw materials of bulk carbon, encompassing the *top-down* approaches to their synthesis. These techniques include laser ablation, arc discharge, chemical oxidation, ball milling, combustion, plasma breakdown, and sono-chemical breakdown. The *bottom-up* approaches refer to the building-up of CQDs from their molecular precursors during chemical reactions. These methods include solvo-thermal synthesis, microwave-assisted synthesis (MWAS), sono-chemical synthesis, thermal decomposition, reverse micelles synthesis, solid state reactions, and electro-oxidation. Both the top-down and bottom-up approaches are summarized in Figure 3.

### 2.1. Top-Down Approaches

#### 2.1.1. Laser Ablation

**Figure 4:**
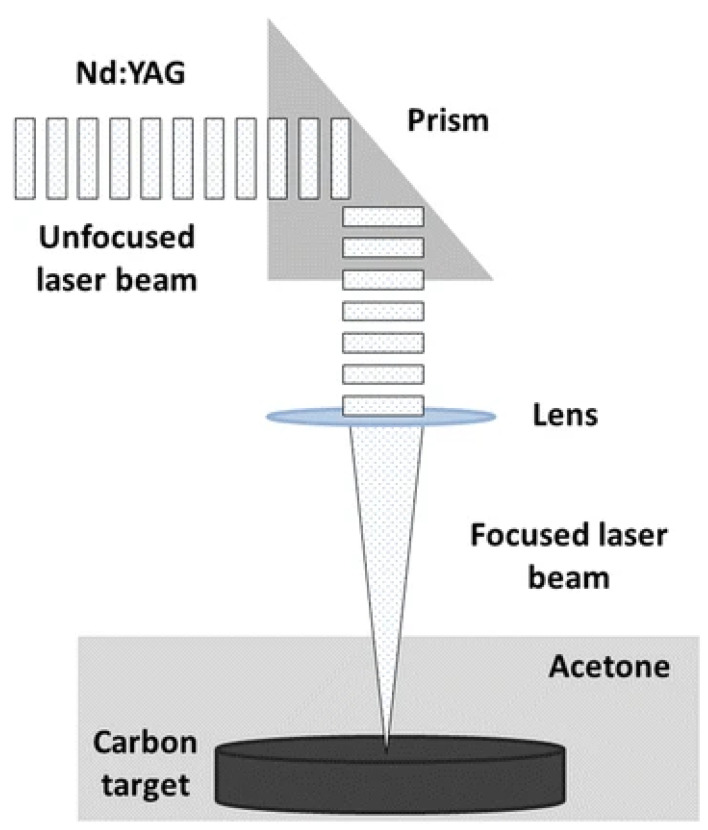
Laser ablation of carbon targets for CQD synthesis. Reprinted with permission from reference [32]. Copyright 2011 Royal Society of Chemistry.

The laser ablation method uses a high-power laser to ablate a bulk carbon target in the presence of a controlled reactive environment [27]. The high-intensity laser is focused on the target to excite high-temperature plasma plumes. The temperature of the plumes can reach up to 100,000 K, depending on the power of laser. This causes the resulting plasma of ultrahigh kinetic energies to expand and cool down, forming a thermodynamic equilibrium. High-energy electrons can then be captured into electronic orbitals of lower energies, simultaneously emitting photons and reducing the temperature. This eventually leads to the formation of CQDs. This technique was conducted utilizing argon and water vapors, which yielded highly stable, 5 nm CQDs with surface passivation [8]. Carbon materials in nitric acid were used as targets for UV laser ablation in the presence of poly-ethylene glycol (PEG) and N-acetyl-L-cysteine (NAC), which also yielded stable surface-functionalized CQDs [28]. The ablation of exfoliated graphite in ionic liquids for CQD synthesis has been reported by Castro et al. [29]. Moreover, low-cost carbon cloth [30] and reduced graphene oxide (rGO) [31] have also been used as precursors for CQD synthesis. Figure 4 shows a schematic of the typical laser ablation-based synthesis of CQDS in acetone [32].

#### 2.1.2. Arc Discharge Method

Various carbon nanostructures, including CQDs, can be prepared using this technique, in which a high-voltage arc discharge between carbon electrodes is employed to generate plasma in inert gas or a liquid environment [33,34,35]. The high-voltage arc causes the carbon atoms to vaporize from the graphite into high energy plasma, leading to recondensation into CQDs. The first synthesis of CQDs in an arc discharge apparatus was reported by Xu et al. [36]. They initially aimed to synthesize multi-walled carbon nanotubes, but found that further filtration could yield CQDs. Mujica et al. made a similar attempt to synthesize CQDs in water. The CQDs emitted strong photoluminescence (PL) at around 406 nm and turned out to be effective for conducting in-vitro cellular uptake as possible bio-markers for cell viability monitoring [37].

#### 2.1.3. Chemical Oxidation

In the chemical oxidation method, bulk structures of carbon precursors are made to break down into nano-scaled CQDs when they are treated with powerful oxidizing agents such as sulfuric acid (H_2_SO_4_) or nitric acid (HNO_3_). This process enables additional surface functional groups to be generated, which enhances the hydrophilicity, ability of fluorescence modulation, and reactivity of the CQDs. Qiao et al. applied the oxidation process by etching carbon that was pre-activated with HNO_3_ to prepare CQDs. The surface passivation was done with amine-terminated compounds, leading to enhanced stability and a high PLQY [38]. The synthesis of surface-functionalized CQDs from a precursor of carbohydrates has also been demonstrated by Peng et al. [39]. Moreover, L-ascorbic acid [40], single-walled carbon nanotubes [41], graphite [42], screen printed carbon electrodes [43], and starch [44] were reported as potential precursors for the chemical oxidative synthesis of CQDs.

Besides the above-mentioned methods, the synthesis of CQDs using other top-down techniques such as ball milling [45], plasma treatment [46], and sono-chemical breakdown [47] have been reported. However, these techniques require further optimization for the controllability of the physicochemical properties of the resulting CQDs.

### 2.2. Bottom-Up Approaches

#### 2.2.1. Solvothermal Synthesis

**Figure 5:**
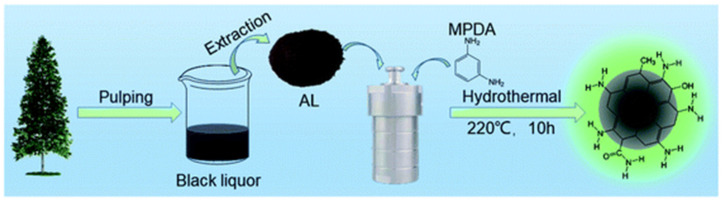
Hydrothermal synthesis of CQDs from alkali lignin obtained from spruce tree for formaldehyde detection. Reprinted with permission from reference [48]. Copyright 2024 Royal Society of Chemistry.

The solvothermal process involves various solvent media and organic precursors, which are carbonized into CQDs under high pressures. The method could be regarded as *hydrothermal* if the solution media were to consist primarily of water. Figure 5 represents a schematic of the hydrothermal synthesis of CQDs from alkali lignin [48]. The solution is typically sealed in an autoclave and kept for a certain period of time inside a heated oven. Zhu et al. used three separate solvents (water, ethanol, and DMF) and synthesized a variety of surface-passivated CQDs using citric acid and urea as reactants at 180 °C for 8 h in an autoclave. The produced CQDs emitted fluorescence of different wavelengths, with PLQYs of up to 47% [14]. Under the same ambient condition, using an aqueous solution of malonic acid and ammonium acetate, CQDs were produced which had size of about 2 nm and a PLQY exceeding 75% [18]. Similarly, Shoujun Zhu et al. achieved a CQD PLQY of 80% [49] using the hydrothermal approach. Recently, Wenjun et al. hydrothermally synthesized CQDs that had controlled red, blue, and green emissions with a color rendering index of 95.4 [50]. It could be concluded that CQDs prepared within hydro/solvo-thermal conditions exhibit strong durability due to having reduced structural defects and surface functionalities as well as adaptability/tunability for use in fluorescence applications in spite of the cost-effectiveness of their fabrication.

#### 2.2.2. Microwaves-Assisted Synthesis

**Figure 6:**
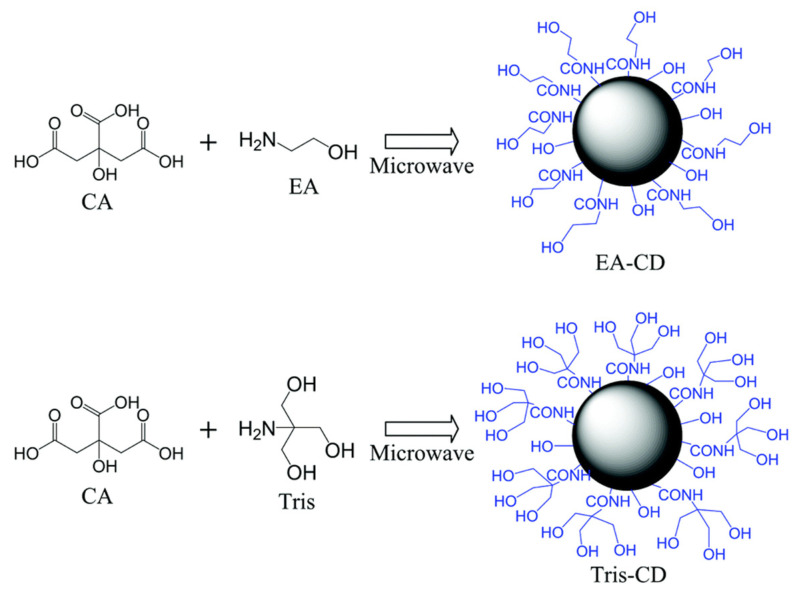
Surface-functionalized N-doped CQDs synthesized by one-step microwave method. Reprinted with permission from reference [7]. Copyright 2009 Royal Society of Chemistry.

Unlike conventional methods that rely on slow heating, microwave-assisted synthesis consists of the rapid heating of the precursor compounds through microwaves for the instant carbonization and surface functionalization of CQDs [51]. Macairan et al. used formamide and glutathione precursors to synthesize water-dispersible, surface-functionalized CQDs under microwave irradiation for 5 min at 180 °C [52]. This method, with the use of succinic acid and tris(2-aminoethyl)amine, could yield CQDs with a PLQY of 49.9% [53]. Zheng et al. synthesized N-doped CQDs with a PLQY of 93.3% using citric acid and tris(hydroxymethyl)aminomethane (Tris) as carbon and nitrogen sources, respectively [54]. Particularly, CQDs synthesized from citric acid, ethanolamine, and tris(hydroxylmethyl)aminomethane (Figure 6) turned out to produce a PLQY of 99%, the highest PLQY ever reported for CQDs to date [7]. The MWAS-based approach operates at a relatively low temperature and relies on the consumption of a relatively low amount of energy that takes place instantaneously, making it a reliable method for the synthesis of nanomaterials.

#### 2.2.3. Thermal Decomposition

Thermal decomposition, also known as *thermal pyrolysis*, relies on intense heating and a series of processes including dehydration, polymerization, and carbonization. The reaction occurs at high temperatures, at which organic precursors undergo structural rearrangements to form carbonized nano-scaled CQDs in open vessels. Dager et al. conducted the green synthesis of CQDs, i.e., using fennel seed extracts subjected to thermal pyrolysis, and reported a PLQY of 9.5% at an excitation wavelength of 260 nm [55]. Dong et al. also achieved a 9% PLQY using citric acid as a precursor for CQD synthesis [56]. Guo and his co-researchers synthesized CQDs that had green light emission with a PLQY of up to 73% using cyanamide (CH_2_N_2_) as a nitrogen source. CH_2_N_2_ was made to react with citric acid with varying molar ratios (from 0.5 to 8) in de-ionized (DI) water in an open vessel at 220 °C for 5 h [57]. Surface-passivated CQDs with a PLQY of 15.4%, synthesized by pyrolyzing citric acid, were also reported [58]. Prospectively, thermal decomposition could be a potential synthesis route to produce high-quality CQDs with tailored characteristics such as a controlled size, high purity, strong and stable photoluminescence, and surface functionalization. Commercially available, inexpensive organic precursors such as glucose and citric acid can be utilized for CQD synthesis without requiring complex apparatuses.

#### 2.2.4. Sono-Chemical Synthesis

**Figure 7:**
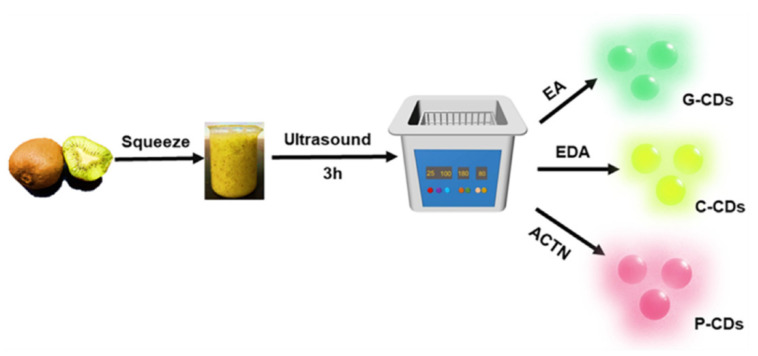
Sono-chemical synthesis of CQDs for luminescence of various colors. Reprinted with permission from reference [61]. Copyright 2022 Nanomaterials.

The sono-chemical approach uses ultrasonic waves to create microscopic bubbles in a precursor solution, thus generating a high local temperature in the solution. This eventually leads to instant CQD formation via rapid energy release by the imploding cavities. Haitao et al. demonstrated the synthesis of 5 nm CQDs from glucose, which had a photostability of over six months and exhibited a PLQY of 7% [59]. Meanwhile, the sono-chemical degradation of PEG CQD formation was also demonstrated. The PEG-coated CQDs were observed to have varying degrees of fluorescence depending on the sonication time and synthesis temperature. A maximum PLQY of 16% was achieved without significant photobleaching after continuous excitation for 10 h [60]. The sono-chemical method could benefit particularly from the doping of impurities. Various colors of luminescence emitting from CQDs, such as chartreuse and pink, can be realized via the use of additives such as ethyl acetate (EA), ethylene diamine (EDA), and acetic acid triethylene ammonium (ACTA) [61]. The sono-chemical synthesis method with respective additives is illustrated in Figure 7.

Other miscellaneous approaches for the synthesis of CQDs include reverse micelles (PLQY of 35%) [62], plasma treatment (PLQY of 9.9%) [63,64], electro-oxidation (PLQY of 0.012%) [65], combustion [66], solid-state reaction (PLQY of 46.4%) [67], and thermal oxidation (PLQY of 01–37%) synthesis [68]. For the optimal synthesis of CQDs, it is recommended to apply the solvo-thermal, MWAS (bottom-up approach), and chemical oxidation methods (top-down) due to their high quantum yields, their compatibility with cost-effective and diverse precursors, and their scalability for industrial production. However, the choice of synthesis method is dependent on the required properties of the CQDs. Table 1 shows a list of synthesis methods and the corresponding PLQYs.

## 3. Physicochemical Characteristics of CQDs

### 3.1. Excitation Dependent Optical Properties of CQDs

**Figure 8:**
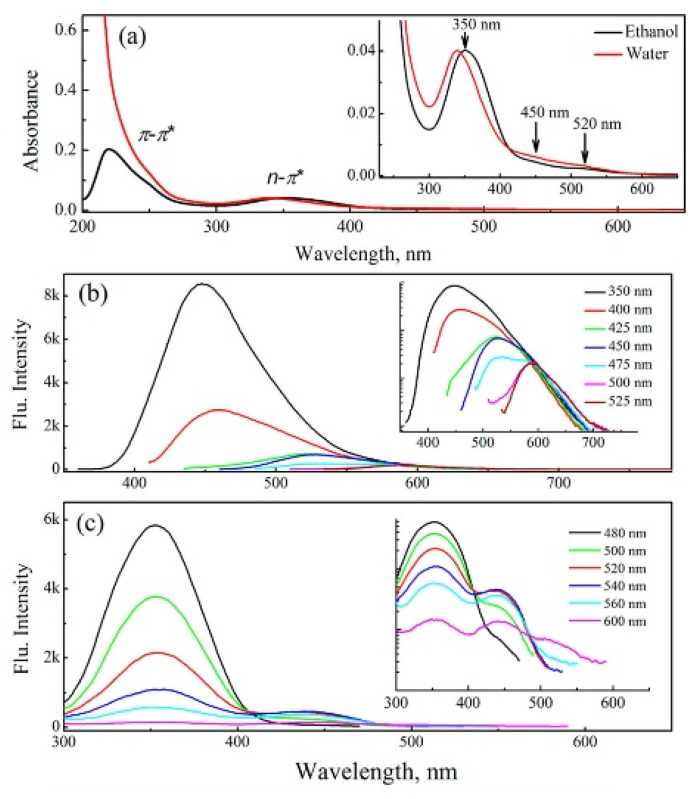
(**a**) Steady-state absorption, (**b**,**c**) emission spectra of CQDs in ethanol for various excitation wavelengths. Reprinted with permission from reference [71]. Copyright 2016 American Chemical Society.

CQDs are garnering attention due to their unique characteristics such as their excitation-dependent luminescence, high quantum yields, and exceptional stability, although these depend on the synthesis method and precursor compounds used. CQDS have the exceptional characteristic of adjusting their fluorescence with varying excitation wavelengths. Figure 8a shows the absorbance spectra of CQDs, revealing π–π* transitions with a peak around 220 nm in sp^2^ carbon domains and n–π* transitions at 340–350 nm from non-bonding electrons in the attached functional groups [71]. The excitation-dependent emission of CQDs is shown in Figure 8b. The ground state shifts into multiple discrete states due to perturbation driven by surface functional groups, generating, namely, ground state heterogeneity. This eventually causes the emission spectrum to redshift as the excitation wavelength changes to a longer wavelength, from 350 to 525 nm. This behavior is possibly due to the formation of J-type aggregates that lead to lower-energy transitions, with a consequence of photon emissions of larger wavelengths [72]. In contrast, the H-type particle aggregates cause a blueshift due to their face-to-face stacking, which resulted in high-energy transitions and ultimately emitted photons of short wavelengths. Figure 8c shows the emission spectra for various excitation wavelengths. These overlap with the absorption spectrum in Figure 8a, confirming the involvement of n–π* and π–π* electronic transitions in the generation of the fluorescence.

**Figure 9:**
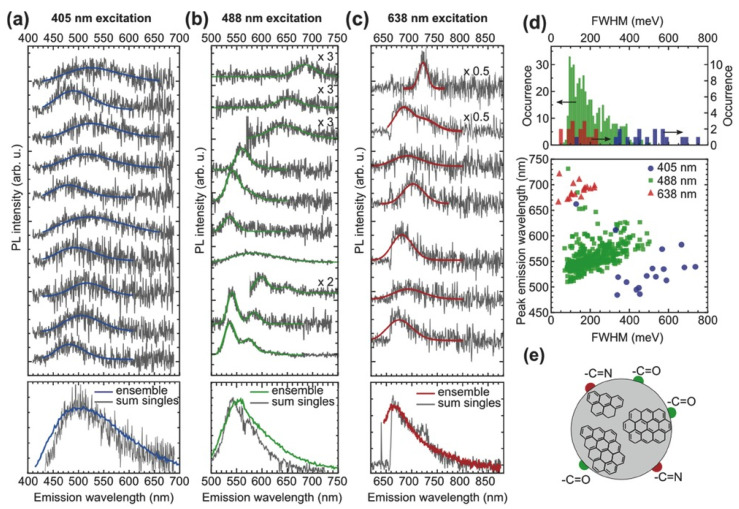
CQDs PL spectra at 405 nm (**a**), 488 nm (**b**), 638 nm (**c**); FWHM distruibution (**d**) and proposed CQD structure (**e**). Reprinted with permission from Ref. [73]. Copyright 2017 SMALL.

The emission spectra of single-CQDs and their ensemble were examined, along with their excitation-dependent emission properties, by Dam et al. [73]. The single-CQD emission spectra reveal distinct states, as seen in Figure 9a–c. The peaks of shorter wavelengths were attributed to sp^2^ domains, while the peaks of the longer wavelengths to domains of carbonyl and imine functional groups. The superposition of the single-CQD emission spectra resulted in a broader spectrum, as shown in “sum singles” in the lower panel of Figure 9a–c. It was also demonstrated that different excitation energies produced different emissive centers in the CQDs (Figure 9d), confirming the role of ground-state homogeneity. The schematic of a CQD in Figure 9e shows the specific link of their structure with their emissions properties, manifesting the interplay of the surface states and CQD cores in the tuning of their optical properties. These features were relevant to the formation of H- and J-type aggregates and to the heterogeneities of the intrinsic ground states of CQDs, contributing to the excitation wavelength-dependence of their emissions spectrum. It was seen that these properties could be exploited to enhance the tunability of fluorescence of CQDs.

### 3.2. Biocompatibility of CQDs

The need for fluorescent materials for biosensing [74], fluorescence probing [75], bioimaging [76], drug delivery [77], etc., is inevitable. The fluorophores used in biosensors require efficient absorption and photoemission with a high PLQY without significant photobleaching [78]. Such demands have been met by numerous probes such as organic dyes and inorganic/organic quantum dots, including CQDs. Organic dyes are easily conjugated with biomolecules and offer relatively high PLQYs [79,80,81]. However, they are known to be generally toxic and hazardous to the ecosystem [82,83,84,85] and suffer quite often from photobleaching [86,87,88], which prevents them from being used in high enough concentrations or under excitation sources with high enough intensity. A detailed description of the hazards of organic dyes has already been published by Tkaczyk et al. [79,80,81,89].

**Figure 10:**
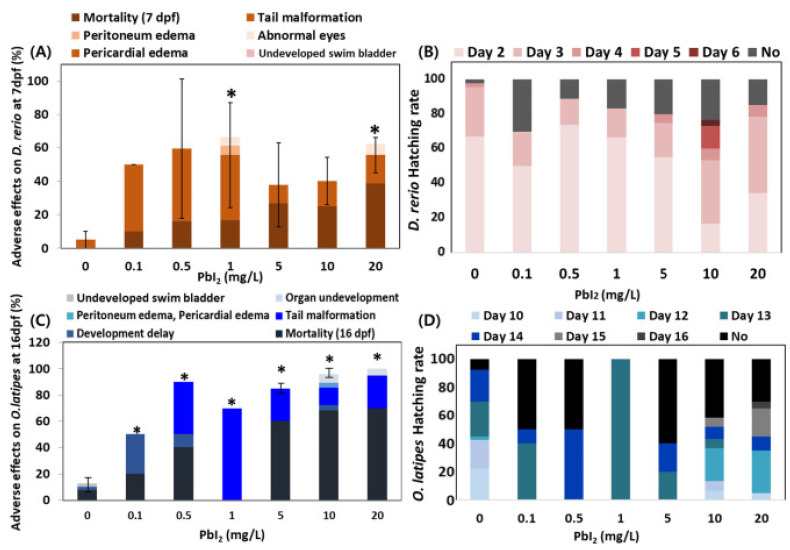
Statistics of the effects of PbI_2_ on (**A**) zebrafish embryos (at 7 days post-fertilization) and (**C**) Japanese medaka embryos at 16 days as well as their hatching rates ((**B**) and (**D**), respectively). PbI_2_ exposure results in developmental failure and an atypical appearance of embryos. The differences between controlled and exposed groups (*p* < 0.05) are indicated with asterisks. Reprinted with permission from reference [92]. Copyright 2021 Elsevier.

Perovskite quantum dots (PQDs) are another promising class of luminescent ceramic nano-particles with a wide absorption spectrum, spanning between infrared and UV regions, and a luminescence spectral window that covers infrared and visible wavelengths [90]. PQDs have gained popularity due to their straightforward synthesis, high PLQY [76], excellent brightness with high color purity, and long exciton lifetime. PQDs have found plenty of applications in solar cells, photodetectors, and LEDs. However, their constituent elements, such as lead (Pb) ions and methylamines, prove toxic and lethal to living cells and the ecosystem, for instance causing neurological diseases and cancer [91]. Figure 10 shows statistical data of the adverse effects of PbI_2_ on zebra fish and Japanese medaka embryos at 7 days post-fertilization [92]. Abnormal appearances and developmental failures were observed in the experimental results. Such harmful effects underscore the need for safer alternatives to methylamine and Pb for use in perovskite compositions. Their lack of biocompatibility makes PQDs inappropriate for use as secure probes or as nano-particles for use in biomedical applications including bioimaging, biosensing, and therapeutic probing.

**Figure 11:**
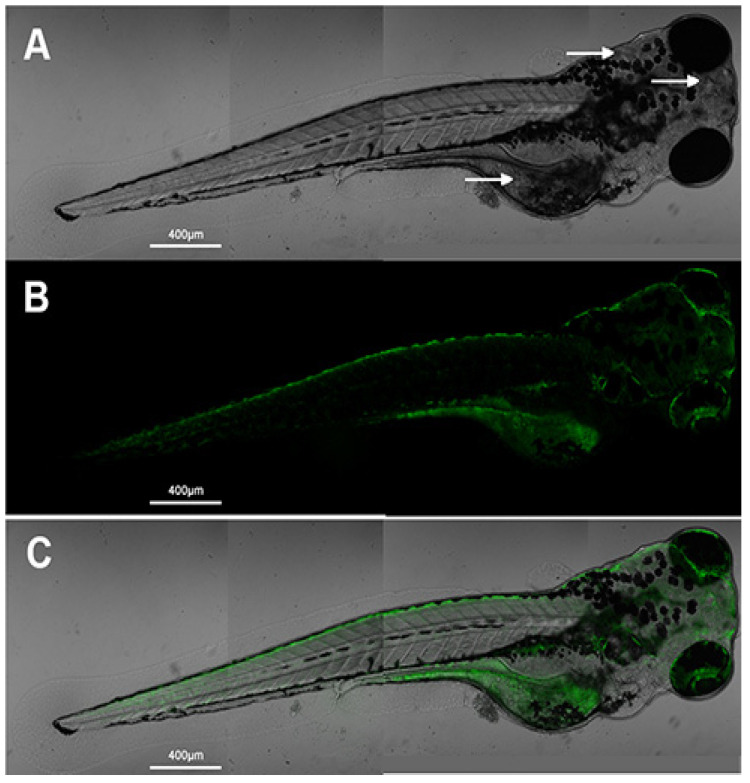
(**A**) Bright-field image of zebra fish incubated with CQDs, (**B**) fluorescence image and (**C**) merged image of a zebrafish incubated with CQDs at λ_max_ = 488 nm [93]; *Int. J. Nanomed.* **2020**, *15*, 6519–6529. Originally published by and used with permission from Dove Medical Press Ltd.

Unlike other quantum dots that frequently pose hazardous threats to the ecosystem and living cells, CQDs are considered non-toxic and biocompatible with living cells while still meeting requirements such as those for luminescent probes: high PLQY, easy synthesis, availability of various surface functional groups, and spectral tunability of luminescence. To the best of our knowledge, no conclusive evidence about the carcinogenicity of CQDs has been reported, as cell viability studies have shown negligible cytotoxicity. Tian et al. reported, in their detailed apoptosis assays and cytotoxicity study, that no adverse effect of CQDs was observed on cell viability. The cellular uptake retained the membrane integrity while the fluorescence of the CQDs remained stable. Their in-vivo application on zebra fish also showed nearly uniform fluorescence without any morphological changes in the tissue of cells observed by confocal microscopy. Figure 11A–C shows bright-field, fluorescence, and merged images of zebra fish with CQD incubation. The uniform green fluorescence indicated that CQDs could be an excellent choice for fluorescence tagging [93].

Ray et al. also reported that CQDs showed virtually no cytotoxicity at even a higher concentration than that required for imaging. Surprisingly, the cell survival rate remained over 90% at a concentration of 0.5 mg/mL [94]. Similar results were obtained [95] when exposing HeLa cells to CQDs at a concentration of 0.08 mg/mL for 36 h. The HeLa cells remained viable, with no apparent damage. Meanwhile, a study on the cytotoxicity of surface-functionalized N-doped CQDs reported a dose-dependent effect on various cell lines; a high concentration of the CQDs produced cytotoxicity while a lower concentration showed negligible toxicity [96]. In summary, it was found that CQDs could be used as eco-friendly, non-toxic (concentration is adjustable to obtain non-toxicity, at least), and safe alternative luminescent probes compared to other harmful photoluminescent materials that are used for applications requiring biocompatibility.

## 4. Fluorescence Quenching Effects in CQDs and Their Utility in Biosensors

**Figure 12:**
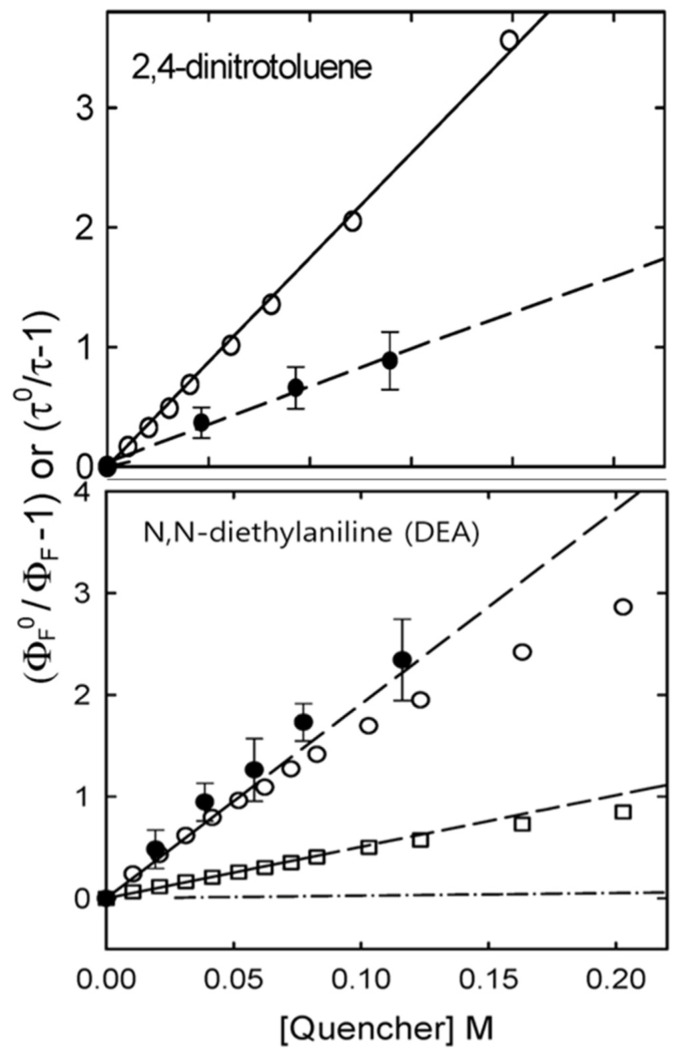
Stern–Volmer plots for the fluorescence quenching of CQDs by 2,4-dinitrotoluene at 425 nm (○) and 407 nm excitation (●) (upper panel) and by DEA at 400 nm excitation in methanol (○) and chloroform (□) and at 407 nm excitation in methanol (●) (lower panel). The low-concentration portion of the same plot for DEA-induced quenching in methanol (-·-) is shown for comparison. Solid and dashed lines are the best fit of the respective data. Reprinted with permission of minor edition from reference [27]. Copyright 2009 Royal Society of Chemistry.

One of the major limitations that CQDs have faced is the fluorescence quenching effect. The fluorescence quenching effect refers to the loss of or reduction in the fluorescence of CQDs when quencher materials interact with them. Such phenomena occur due to the presence of recombination centers in the CQDs, through which non-radiative decay occurs by way of mid-gap states within the band-gap energy. This process mitigates the chances of electrons recombining with holes for fluorescence generation, instead converting excitation photon energy into heat. In many cases, the photoexcited carriers (electrons and holes) transfer to the adjacent molecules/atoms and, hence, prevent the radiative recombination. In CQDs, *static quenching* may occur due to the creation of a ground-state complex between the CQDs and the quencher material, which would not support radiative decay. Meanwhile, *dynamic quenching* may result from the non-radiative deactivation of CQDs due to them colliding with quencher molecules. Xin Wang et al. presented the Stern–Volmer measurements to explain the fluorescence quenching in CQDs synthesized by laser ablation (Figure 12) [27]. Quenching basically takes place through the transfer of photo-excited carriers, whereby CQDs play roles as acceptors or donors of electrons with neighboring quencher molecules/atoms. In other words, quencher molecules can be acceptors or donors, contributing to the quenching of the fluorescence of CQDs, for example N,N-diethylaniline and 2,4-dinitrotoluene. Static quenching can occur when noble metals such as platinum (Pt) and gold (Au) are deposited on the surfaces of CQDs, where metal salts (ions) cause the deactivation of radiative decays. Such deactivation of exciton radiative decays occurs primarily in local regions around the surface trapping sites of CQDs [97]. Hg(II) and Cu(II) negatively affect the fluorescence of N-acetylcysteine (NAC)-functionalized CQDs via the formation of a stable complex between NAC sulfur groups and Hg(II), with a quenching constant (*K_sv_ = 1.3 × 10⁵*) being observed through Stern–Volmer analysis.

Iron ions (Fe^3+^) highly inhibit the fluorescence of CQDs by receiving photo-excited electrons from them. A negative linear relationship between the fluorescence intensity ratio and the Fe^3+^ ion concentration has been reported by Khan et al. [67]. The combined effects of the transfer of photo-excited electrons [27] and the complex formation between CQDs and metal ions (Fe^3+^, Cu^2+^ and Mn^2+^) used for quenching have been investigated by Guo et al. [57]. Such responses of CQDs make them ideal for use in rapid and highly sensitive optical biosensors for the selective monitoring of metal ions in biological fluids under UV light illumination. CQD fluorescence quenching could find applications in water quality testing and environmental monitoring. The pH-dependent fluorescence of CQDs and their high photostability make them suitable for use in cellular pH sensing optical biosensors [38]. The reduction of luminescence of CQDs, which was shown to be linear with the concentration of Hg^2+^, could find use in the environmental monitoring of mercury contamination [18]. The fluorescence of nitrogen-doped functionalized CQDs was shown to be prone to quenching selectively only when interacting with dopamine. This was due to the electrostatic interaction between the surface functional groups of CQDs and the amino groups of dopamine molecules. These characteristics of CQDs could lend themselves to use as a biosensor that could sense dopamine in biological fluids such as human blood [98].

In contrast to the abovementioned quenching phenomena, metals such as Ni(II), Zn(II), Cd(II), and Ca(II) ions did not show any measurable quenching effects. Interestingly, the fluorescence of PEG-only functionalized CQDs remained unaffected by Hg(II) and Cu(II) [28]. This could be due to the formation of a protective layer around the CQDs that isolates their emissive core from the surrounding quencher molecules. Other possibilities are the reduction in surface defects and the extension in spatial separation between the CQDs and the quencher caused by PEGylation that blocks the pathways for electron transfer, leading to stable fluorescence.

Other factors could also contribute to the fluorescence quenching of CQDs, such as surface defects and aggregation. Unwanted defect (electronic) states of sufficiently high density could trap photo-excited carriers, strengthening non-radiative decays. In addition, the aggregation of CQDs could induce non-radiative decays of photo-excited carriers via inter-core π–π orbital interactions and thus facilitate the energy loss mechanism. The aggregation-based quenching of CQDs could be utilized for the selective detection of various analytes; for example, nitrogen and sulfur co-doped CQDs can quantitatively detect quercetin in real food samples [23], 4-nitrophenol [24] in water, and propranolol in plasma [22]. The detection of Cr(VI) ions in aquatic eco-systems through an aggregation-based quenching effect in CQDs has also been reported [99]. Solvo-thermally synthesized yellow emissive CQDs can quantify bilirubin in human urine and sera [25]. This mechanism can even find application in hypersensitive optical biosensors with rapid and real-time detection for the quantitative analysis of specific analytes through Stern–Volmer plots or similar standardization curves. In the next subsection, we deal with the strategies for how to mitigate the environmental and non-selective quenching effects of CQDs.

## 5. Mitigating Fluorescence Quenching for Optoelectronic Devices

Solid state CQDs often suffer from fluorescence quenching due to π–π orbital stacking, non-radiative energy transfer, and aggregation, as mentioned above [23,24,57,67,98,99]. However, they may be reactivated to fluoresce under UV light excitation in solvents like water, dimethyl sulfoxide (DMSO), N,N-dimethylformamide (DMF), ethanol, etc. [57]. Various strategies have been reported to mitigate the fluorescence quenching in solid state CQDs. For example, the crosslinking of polymers and alkane chains could facilitate the steric hinderance, preventing quenching [100,101]. Embedding CQDs in salt citrate crystals could also inhibit quenching [102,103]. CQD aggregation, one of the major causes of fluorescence quenching, could be avoided by introducing electrostatic repulsion and hydrogen bonding [104]. Moreover, nanoparticle size uniformity has been considered to suppress non-radiative energy transfer [105].

The post-synthesis treatment of CQDs with polymer matrices like PVA, PEG, and PMMA would increase the inter-particle separation and provide steric hinderance for the mitigation of fluorescence quenching [106]. Doping heteroatoms into CQDs (such as Si, B, and N), salt embedding, and reducing the number sp^2^ regions enhanced the PLQY of CQDs with multi-color wavelengths [107,108]. Table 2 summarizes various strategies to circumvent fluorescence quenching in CQDs, aiming to enhance their PL properties.

## 6. Conclusions

CQDs have emerged as promising nano-probes for fluorescence, benefitting from their easy fabrication, eco-friendly synthesis, potential scalability for mass production, availability of versatile surface functional groups, considerably high PLQY, wide spectral window for excitation and emission, spectral tunability of luminescence, and biocompatibility. CQDs can lend themselves to use in biosensors, bio-imaging, and therapeutics. Their synthesis methodologies are subgrouped into bottom-up and top-down approaches. The bottom-up approaches include the solvo-thermal method and MWAS, while the top-down approaches include laser ablation and chemical oxidation. CQDs could effectively detect biochemical molecules such as dopamine, reactive oxygen species, and metal ions for selective analysis. The current advancements in their synthesis strategies, doping, and surface functionalization will be able to be further optimized CQDs for bio-sensing/bio-imaging applications that require the sustainable and scalable integration of CQDs.

### Future Research Directions

Future research should focus on enhanced control over CQD properties for further advancement in their applications. Precise methods for CQDs synthesis need to be developed to control for their size, surface functionalities, cost, reproducibility, and scalability. Further improvement in their PLQY and photostability still remain necessary. Doping CQDs with external atoms and molecules can be one of the pathways towards their effective improvement while maintaining their intrinsic biocompatibility.

## Figures and Tables

**Table 1:** A list of CQD synthesis methods with respective precursors and PLQYs.

CQDs Synthesis Method	PLQY (%)	Precursor	Refs.
Laser Ablation	35.4	Bulk Carbon/Graphite	[8,27,28,29,30,31]
Arc Discharge	16	Bulk Carbon/Graphite	[33,34,35,36,37]
Chemical Oxidation	12.7	Graphite, Carbohydrates	[38,39,40,41,42,43,44,69]
Ball Milling	74.55	Bituminous Coal	[45]
Plasma Treatment	1.6	Isopropanol	[46,70]
Sono-chemical	7	Glucose	[47]
Solvothermal synthesis	96.12	PEG, RhB	[14,18,49,50]
Microwaves assisted	99	Citric acid, Amino compounds	[7,51,52,53,54]
Thermal decomposition	73	Cyanamide	[55,56,57,58]
Sono-chemical synthesis	16	PEG	[59,60,61]
Reverse micelles	35	Glucose	[62]
Electro-oxidation	0.012	Graphite	[65]
Combustion	0.8	Candle soot	[66]
Solid state reaction	46.4	Citric acid, diammonium citrate	[67]
Thermal oxidation	3	Citric acid	[68]

**Table 2:** A list of strategies to mitigate fluorescence quenching in CQDs.

Strategy		Description	References
1	Crosslinking of polymers and alkane chains	Polymer chains provide steric hindrance, preventing fluorescence quenching.	[100,101]
2	CQDs embedding in salt citrate crystals	Inhibits fluorescence quenching by encapsulating CQDs in stable crystals	[102,103]
3	Avoiding CQDs aggregation	Introduces electrostatic repulsion and hydrogen bonding to prevent aggregation, a major cause of quenching	[104]
4	Nanoparticle size uniformity	Uniform size distribution of CQDs suppress non-radiative energy transfer	[105]
5	Treatment of CQDs with polymer matrices	Embedding CQDs in PVA, PEG increases spatial separation and provides steric hindrance to circumvent quenching.	[106]
6	Doping heteroatoms in CQDs structure	Doping heteroatoms like B, Si, and N enhances PLQY.	[107,108]

## Data Availability

No new data were created or analyzed in this study.
